# Mediating mechanisms of the association between prenatal tobacco exposure and adolescent suicide attempt: A 17-year longitudinal study

**DOI:** 10.1017/S0033291725102936

**Published:** 2026-01-02

**Authors:** Audrée Bujold, Kelly Jane Rosialda, Massimiliano Orri

**Affiliations:** 1 McGill Group for Suicide Studies, Douglas Mental Health University Institute, Department of Psychiatry, McGill University, Montreal, Quebec, Canada; 2 Department of Psychology, McGill University, Montreal, Quebec, Canada; 3 Department of Epidemiology, Biostatistics, and Occupational Health, School of Population and Global Health, McGill University, Montreal, Quebec, Canada; 4 Danish Research Institute for Suicide Prevention, Mental Health Centre Copenhagen, Copenhagen, Denmark

**Keywords:** development, longitudinal study, maternal smoking, mediation analysis, suicide attempt

## Abstract

**Background:**

While prenatal exposure to tobacco has been associated with adolescent suicide attempt, little is known about the mechanisms explaining this association. This study aims to explore the mediating roles of internalizing symptoms, externalizing behaviors, and peer problems across childhood in the association between prenatal exposure to tobacco and adolescent suicide attempt.

**Methods:**

We analyzed data from *N* = 8,861 participants from the Millennium Cohort Study followed from ages 9 months to 17 years. Binary logistic regression models were used to investigate the total association between exposure to tobacco in pregnancy and suicide attempt, and mediation analyses were conducted using structural equation models to investigate the direct and indirect associations.

**Results:**

In models adjusted for key covariates, we found a significant association between prenatal tobacco exposure and increased risk of adolescent suicide attempts (odds ratio = 2.08, 95% confidence interval = [1.68, 2.56]), partly mediated through internalizing problems, externalizing behaviors, and peer problems from ages 3 to 14 years (accounting for 37% of the total association, that is, 16%, 12%, and 9%, respectively).

**Conclusions:**

These findings suggest that interventions targeting mental health symptoms and peer problems may maximize suicide prevention efforts among children who were prenatally exposed to tobacco, thus potentially reducing the long-term risk of suicide attempt.

## Introduction

Adolescent suicide is an important public health concern, representing one of the leading causes of death worldwide, with increasing rates in the past years in several countries (Bertuccio et al., 2024). To develop effective early intervention strategies, it is essential to understand the underlying risk factors associated with adolescent suicidal behaviors (Bostwick, Pabbati, Geske, & McKean, 2016). While research has largely investigated proximal risk factors of suicide, such as psychiatric disorders, an increasing body of evidence highlights the long-term impact of early-life (i.e. perinatal and early childhood) exposures on suicide risk (Orri et al., 2019).

Among the various prenatal factors investigated in the literature, maternal smoking during pregnancy has emerged as a potential risk factor for a range of adverse developmental and psychological outcomes in the offspring (Corrêa et al., 2022). Nicotine, the primary psychoactive component of tobacco, has been shown to cross the placenta, enter the fetal circulation, and influence the fetus in a critical moment of brain development (Ekblad, Lehtonen, Korkeila, & Gissler, 2017), thus potentially increasing the risk of developing mental disorders. Some studies have suggested that exposure to tobacco during pregnancy may also increase the risk of suicide attempts in adolescence (Vidal-Ribas, Govender, Sundaram, Perlis, & Gilman, 2022).

Although the causal role of prenatal tobacco exposure on adolescent suicide attempt has not been definitively established (Alaräisänen et al., 2012; Orri et al., 2021), prevention of in-utero exposure to tobacco may yield positive effects in terms of preventing future risks of mental health problems, including suicidal behaviors. In this perspective, it is important to understand the mechanisms explaining the link between prenatal tobacco exposure and adolescent suicide attempt, as identifying such mechanisms can help us identify potential intervention targets for children prenatally exposed to tobacco.

Maternal smoking during pregnancy has been associated with several behavioral, social, and emotional problems during early and middle childhood. For example, studies have shown an increased risk of internalizing symptoms during childhood among children exposed to tobacco in utero (Moylan et al., 2015). This exposure has also been associated with an increased risk of inattention, hyperactivity, and behavioral problems in childhood and adolescence (Godleski, Shisler, Colton, & Leising, 2024; Roza et al., 2009), with evidence of dose–response relationships (Brennan, Grekin, Mortensen, & Mednick, 2002). Moreover, studies have shown that being exposed to tobacco during pregnancy was associated with a higher likelihood of experiencing difficulties in social relationships across childhood (Brion et al., 2010; Sutin, Flynn, & Terracciano, 2017). These behavioral and psychosocial difficulties may heighten the risk of developing suicidal behaviors (Geoffroy et al., 2016; Min et al., 2021; Soto-Sanz et al., 2019; Van Meter, Paksarian, & Merikangas, 2019; Wei et al., 2016).

However, to our knowledge, no prior studies have investigated the potential mediating roles of internalizing symptoms, externalizing behaviors, and peer problems in the association between prenatal tobacco exposure and risk of adolescent suicide attempts. Identifying these psychosocial pathways is crucial, as it could help inform the implementation of existing evidence-based interventions that address such difficulties in children exposed to tobacco during pregnancy, ultimately reducing suicide risk and improving the well-being of these individuals.

Using data from a 17-year population-based cohort, this study aims to investigate the potential mediating roles of internalizing symptoms, externalizing behaviors, and peer problems during childhood and adolescence in the association between exposure to maternal smoking during pregnancy and offspring suicide attempt at age 17 years. We hypothesize that prenatal tobacco exposure may increase vulnerability to emotional difficulties (e.g. anxiety and depression), contribute to behavioral dysregulation and impulsivity, and lead to challenges in forming and maintaining positive peer relationships across childhood, which together may heighten the risk of suicide behaviors during adolescence (Gunzler, Chen, Wu, & Zhang, 2013). As such, we expect that the association between maternal smoking and offspring suicide attempt will be partially mediated by internalizing symptoms, externalizing behaviors, and peer problems.

## Methods

### Participants

Participants were drawn from the Millennium Cohort Study (MCS), a nationally representative prospective cohort including 18,827 children born in the United Kingdom between September 2000 and January 2002. Families receiving Child Benefit (including 98% of the population at the time of sampling) were randomly selected from electoral wards using a stratified sampling design. This ensured adequate representation across all United Kingdom countries, as well as disadvantaged and ethnically diverse areas (Connelly & Platt, 2014; Joshi & Fitzsimons, 2016). The initial wave of data collection took place when cohort members were 9 months old, followed by additional waves at ages 3, 5, 7, 11, 14, and 17 years. Our analyses included 8,861 participants with available data on maternal smoking during pregnancy and suicide attempt at age 17 years. Included and excluded participants differed on all measured sociodemographic characteristics at baseline (Supplementary Table S1), potentially limiting the generalizability of the findings and representing a source of bias that should be considered when interpreting the results. The MCS received ethical approval from the National Health Service Research Ethics Committee system, which ensured compliance with ethical guidelines for participant safety and consent (Connelly & Platt, 2014).

### Measures

#### Maternal smoking during pregnancy

Mothers self-reported their smoking behavior by answering the following questions when children were 9 months old; ‘About how many cigarettes a day were you smoking just before you became pregnant with [child name]’, ‘And did you change the amount you smoked during your pregnancy?’ and ‘How many cigarettes a day did you usually smoke after you made this change?’ These variables were recoded into a single binary measure, where mothers who reported smoking at any point during pregnancy were classified as having smoked during pregnancy.

#### Mediators

Internalizing symptoms, externalizing behaviors, and peer problems were measured using items from the parent-reported version of the Strengths and Difficulties Questionnaire, a validated scale assessing child and adolescent mental health symptoms (Goodman & Goodman, 2009). Items used for our mediators were collected at ages 3, 5, 7, 11, and 14 years. All items were answered using a 3-point Likert-scale (1 = *Not true*, 2 = *Somewhat true*, and 3 = *Certainly true*), which we standardized (*z*-score transformed) and averaged to calculate final scores at each time point and overall scores. Internalizing symptoms were assessed with the following items: ‘often complains of headaches’, ‘many worries, often seems worried’, ‘often unhappy, downhearted’, ‘nervous or clingy in new situations’, and ‘many fears, easily scared’ (*α* = 0.76–0.82). For externalizing behaviors, the conduct problems subscale, including ‘often has temper tantrums or hot tempers’, ‘generally obedient (reverse-scored)’, ‘often fights with other children’, ‘often lies or cheats’, and ‘steals from home, school or elsewhere’ (*α* = 0.77–0.85), was combined with the hyperactivity subscale, including ‘restless, overactive’, ‘constantly fidgeting or squirming’, ‘easily distracted, concentration wanders’, ‘thinks things out before acting (reverse-scored)’, and ‘seeks tasks through to the end (reverse-scored)’ (*α* = 0.78–0.86). To assess peer problems, the following items were used: ‘rather solitary, tends to play alone’, ‘has at least one good friend (reverse-scored)’, ‘generally liked by other children (reversed-scored)’, ‘picked on or bullied by other children’, and ‘gets on better with adults than with other children’ (*α* = 0.72–0.80).

#### Adolescent suicide attempt

At age 17 years, adolescents were asked ‘Have you ever hurt yourself in an attempt to end your life?’, which was in reference to the past year.

#### Covariates

We selected the following covariates to use in our multivariable models, all of which were reported by the parent when the child was 9 months old. Sociodemographic characteristics included sex (male/female), ethnicity (white/not white), familial income (low: <10,400£/medium: 10,400–31,200£/high: >31,200£, using a variable with net income categories defined by the MCS investigators, which accounts for the presence of one or both parents in the household). Family characteristics included maternal psychological distress (measured using the modified rutter malaise inventory, a validated instrument to measure levels of anxiety and distress (Johnson, Atkinson, & Rosenberg, 2015), maternal age at childbirth (in years), maternal consumption of alcohol during pregnancy (yes/no), presence of caregivers in the household (both parents/blended family or other caregiver), and whether the pregnancy was planned or a surprise.

### Statistical analysis

Main analyses were performed using R version 4.4 and Mplus version 8.4; 95% confidence intervals (CIs) were calculated using robust standard errors (SEs). The computer code used for these analysis is available at https://github.com/OrriLab/smoking-suicide-attempt-mediators

#### Association between smoking during pregnancy and suicide attempt

Binary logistic regression models were first conducted to investigate the associations of maternal smoking during pregnancy with adolescent suicide attempt. The association was expressed as an odds ratio (OR) and represents the *total association* (Preacher & Kelley, 2011). We estimated both unadjusted models and models adjusted for the selected covariates. Sex differences in this association were investigated by testing an interaction between sex and maternal smoking. If statistically significant, primary analyses will focus on sex-specific associations. If not statistically significant, primary analyses will focus on the whole sample, but separate analyses for males and females will be reported as complementary analyses. Similarly, primary analysis will focus on the binary indicator of smoking versus not smoking during pregnancy, but complementary analyses will be conducted to investigate the associations using the number of cigarettes smoked as a continuous variable (standardized into a *z*-score).

#### Mediation analysis

Mediation analyses were performed using structural equation models to examine the *indirect association* of maternal smoking during pregnancy with adolescent suicide attempt through three potential mediators: internalizing symptoms, externalizing behaviors, and peer problems (Gunzler et al., 2013). Indirect associations were calculated using the product of the coefficient approach (i.e. the product of the coefficient for the association between exposure and mediator, and between mediator and outcome). First, each mediator was analyzed separately to assess their individual contribution to the overall relationship, then all mediators were tested in a single model to account for their correlation. Indirect associations for each mediator were thus obtained, representing the part of the total association explained by each of them. The remaining unexplained part was represented by the *direct association* (total association = indirect + direct associations). The proportion of the association mediated was also calculated as the ratio between the indirect association and total association, to better understand the relative importance of each mediator. Both unadjusted and adjusted models were estimated.

#### Missing data

Missing data in the covariates and mediators (Supplementary Figure S1), representing 14.2% of the dataset, were imputed using the *missForest* package in R, a nonparametric method based on random forests, and the imputed dataset was used for all association analyses.

## Results

**Figure 1.**
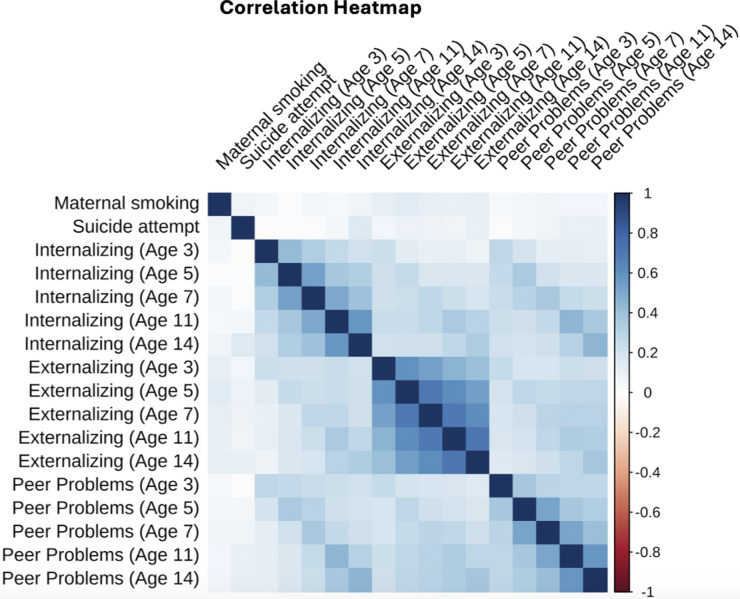
Heatmap of the correlations between the main variables of interest.

The characteristics of the participants in this study are presented in Table 1. Among the 8,861 participants, 4,342 (48.8%) were male and 4,519 (51.2%) were female; 7,160 (81.0%) were White and 1,682 (19.0%) were of any other ethnic minority. Correlations between the main variables of interest (exposure, mediators, and outcome) are presented in Figure 1. The strongest positive correlations were observed within domains across time (e.g. emotional symptoms from age 11 to 14 years: *r* = 0.61), suggesting longitudinal stability.

**Table 1.** Sample characteristics

	Sample (*N* = 8,861)	Male(*N* = 4,342)	Female(*N* = 4,519)	*p*-values
**Child characteristics**				
Ethnicity				
White	7,160 (80.8%)	3,518 (81%)	3,658 (80.9%)	0.95
Non-White (ethnic minority)	1,682 (19%)	824 (19%)	861 (19.1%)	
**Socioeconomic environment**				
Income, *n (%)*				0.52
Low, <10,400£	1,695 (19.1%)	2,394 (55.1%)	2,476 (54.8%)	
Medium, 10,400–31,200£	4,401 (49.7%)	1,069 (24.6%)	1,086 (24.0%)	
High, >31,200£	2,046 (23.1%)	879 (20.2%)	957 *21.2%)	
**Family environment**				
Presence of caregivers in the household				0.34
Both parents	7,816 (88.2%)	3,845 (88.6%)	3,971 (87.9%)	
Blended family or other caregiver	1,045 (11.8%	497 (11.4%)	548 (12.1%)	
**Maternal variables**				
Alcohol consumption during pregnancy				0.25
No	6,146 (69.4%)	2,992 (68.9%)	3,166 (70.1%)	
Yes	2,702 (30.5%)	1,350 (31.1%)	1,353 (29.9%)	
Psychological distress, mean (SD)	1.61 (1.67)	1.66 (1.69)	1.60 (1.65)	0.11
Maternal age, mean (SD)	30.01 (5.72)	30.01 (5.70)	30.02 (5.74)	0.92
Planification of pregnancy				0.75
Planned	5,295 (59.8%)	2,608 (60.1%)	2,698 (59.7%)	
Surprise	3,541 (40%)	1,734 (39.9%)	1,821 (40.3%)	
**Mediating variables**				
Emotional problem score, mean (SD)				
3 years	1.25 (0.26)	1.27 (0.26)	1.27 (0.26)	0.28
5 years	1.26 (0.29)	1.27 (0.30)	1.28 (0.29)	0.10
7 years	1.29 (0.33)	1.31 (0.34)	1.32 (0.32)	0.37
11 years	1.35 (0.37)	1.35 (0.36)	1.38 (0.37)	< 0.001
14 years	1.39 (0.39)	1.34 (0.36)	1.45 (0.40)	< 0.001
Externalizing problem score, mean (SD)				
3 years	1.65 (0.34)	1.69 (0.35)	1.62 (0.33)	
5 years	1.47 (0.31)	1.51 (0.33)	1.42 (0.29)	< 0.001
7 years	1.46 (0.33)	1.51 (0.35)	1.41 (0.31)	< 0.001
11 years	1.43 (0.32)	1.48 (0.34)	1.38 (0.30)	< 0.001
14 years	1.42 (0.32)	1.46 (0.33)	1.37 (0.29)	< 0.001
Peer problem score, mean (SD)				
3 years	1.27 (0.26)	1.32 (0.27)	1.28 (0.26)	< 0.001
5 years	1.20 (0.26)	1.26 (0.27)	1.22 (0.25)	< 0.001
7 years	1.21 (0.27)	1.27 (0.29)	1.22 (0.25)	< 0.001
11 years	1.25 (0.31)	1.29 (0.32)	1.24 (0.29)	< 0.001
14 years	1.33 (0.33)	1.35 (0.34)	1.32 (0.32)	< 0.001

*Note*: *p*-values indicate differences between males and females, and are based on *t*-tests or chi-square tests for continuous and categorical variables, respectively.

### Association between maternal smoking during pregnancy and childhood psychosocial difficulties

A total of 1,229 (17%) children were exposed to tobacco at some point during their mother’s pregnancy. On average, mothers reported smoking 0.91 cigarettes during pregnancy (standard deviation [SD] = 3.22). Children whose mothers smoked during pregnancy were more likely to exhibit internalizing symptoms (*β* = 0.205, SE = 0.031, *p* < 0.001), externalizing behaviors (*β* = 0.477, SE = 0.031, *p* < 0.001), and peer problems (*β* = 0.279, SE = 0.031, *p* < 0.001) across their childhood.

### Association between childhood psychosocial difficulties and adolescent suicide attempt

At age 17 years, 627 (7%) adolescents reported having attempted suicide in the past year. We found that externalizing behaviors (*β* = 0.188, SE = 0.051, *p* < 0.001; OR = 1.21, 95% CI = [1.09, 1.33]) and peer problems (*β* = 0.241, SE = 0.052, *p* < 0.001; OR = 1.27, 95% CI = [1.15, 1.41]) from ages 3 to 14 increased the likelihood of later suicide attempt. Internalizing symptoms were not significantly associated with increased likelihood of suicide attempt (*β* = 0.057, SE = 0.052, *p* = 0.278; OR = 1.06, 95% CI = [0.96, 1.17]).

### Pathways of association between maternal smoking during pregnancy and adolescent suicide attempt

**Figure 2.**
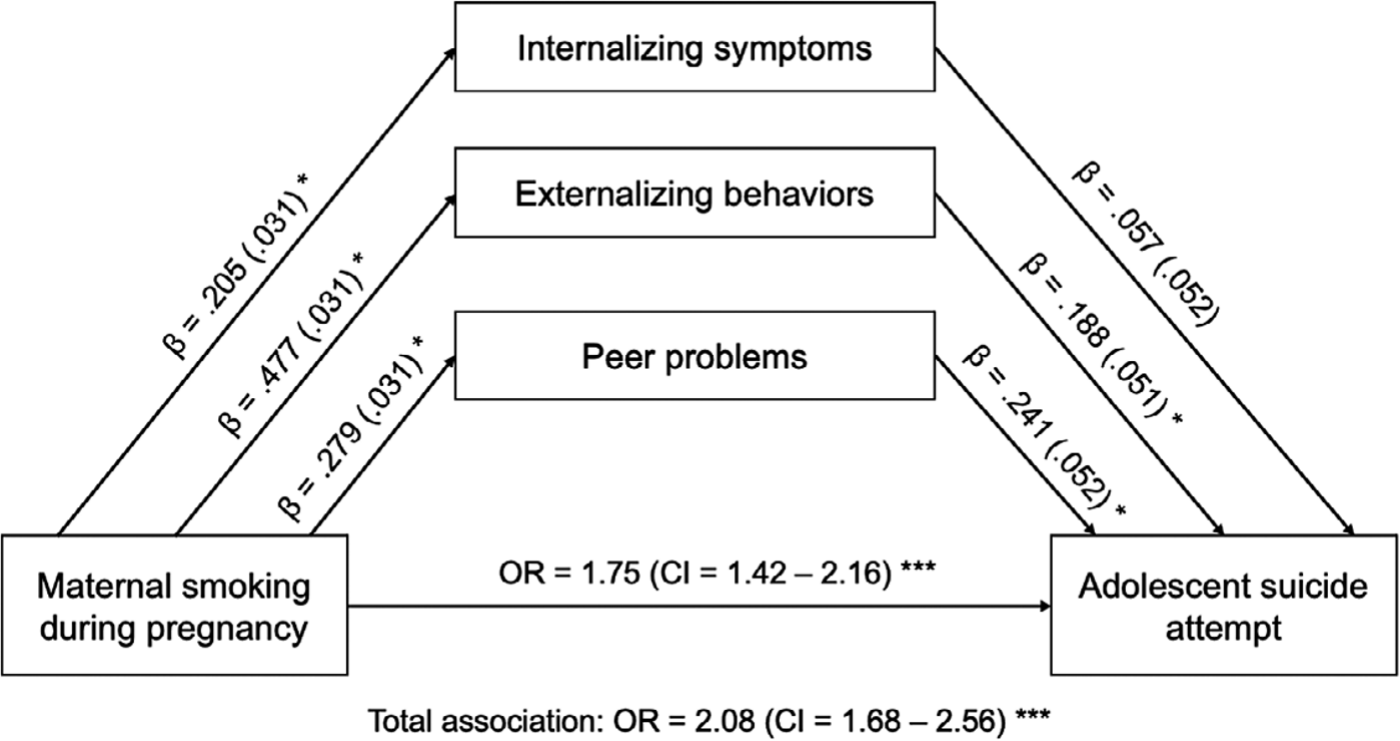
Path analysis model estimating the direct and indirect associations between prenatal exposure to tobacco and adolescent suicide. Associations are reported as beta coefficient (*β*) and standard error, or odds ratios (ORs) and 95% confidence intervals (CIs). *p < 0.001.

Among individuals exposed to tobacco smoking during pregnancy, 167 (26.63%) reported a suicide attempt at age 17 years, compared to 460 (73.27%) individuals not exposed to tobacco smoking during pregnancy (OR = 2.47, 95% CI = [2.04, 2.99]) (Table 2). Suicide attempt was more prevalent among females (*n* = 447, 71.3%) than among males (*n* = 180, 28.7%; *χ*^2^ = 110.32, *p* = 0.00). However, the association between exposure to smoking and suicide attempt was not statistically different for males and females (*p*_interaction_ *=* 0.97). Thus, our primary analyses focused on the full sample. Structural equation models demonstrated that prenatal smoking was significantly associated with an increased risk of suicide attempt in adolescence (total association: *β* = 0.731, SE = 0.107, OR = 2.08, 95% CI = [1.68, 2.56]) after adjusting for the selected covariates. This indicates that individuals prenatally exposed to tobacco had 1.75 times higher odds of attempting suicide compared to those who were not exposed. Mediation analysis showed that 12.3% of the association was explained by externalizing behaviors (indirect association: *β* = 0.90, SE = 0.025, OR = 1.09, 95% CI = [1.04, 1.15]), and 9.2% by peer problems (indirect association: *β* = 0.057, SE = 0.025, OR = 1.07, 95% CI = [1.03, 1.11]). Internalizing symptoms explained 16% of the association, although the indirect association was not statistically significant (*β* = 0.012, SE = 0.011, OR = 1.01, 95% CI = [0.99, 1.03]) (Figure 2). The proportion of the total effect mediated through all three mediators was estimated at 23% (overall indirect effect: *β* = 0.169, SE = 0.023, OR = 1.18, 95% CI = [1.13, 1.24]), suggesting that ~23% of the association between prenatal smoking and adolescent suicide attempts is attributed to these mediators. The direct association of prenatal tobacco exposure on suicide attempts remained significant (*β* = 0.562, SE = 0.107, OR = 1.75, 95% CI = [1.42, 2.16]). That is, even after accounting for mediators, prenatal exposure to tobacco was associated with more than a twofold increase in the odds of attempting suicide (Table 2).

**Table 2.** Total, direct, and indirect associations

	Unadjusted		Adjusted^a^	
	OR	95% CI	OR	95% CI
**Whole sample**				
Total association	2.47	2.04–2.99	2.08	1.68–2.56
Direct association	2.20	1.81–2.67	1.75	1.42–2.16
Indirect association: Internalizing symptoms	1.03	1.01–1.06	1.01	0.99–1.03
Indirect association: Externalizing behaviors	1.05	1.00–1.09	1.09	1.04–1.15
Indirect association: Peer problems	1.04	1.01–1.07	1.07	1.03–1.11
Total indirect association	1.12	1.08–1.18	1.18	1.13–1.24
**Female only**				
Total association	2.58	2.04–3.27	2.23	1.72–2.88
Direct association	2.18	1.72–2.77	1.88	1.45–2.42
Indirect association: Internalizing symptoms	1.02	0.99–1.04	1.01	0.99–1.04
Indirect association: Externalizing behaviors	1.12	1.05–1.18	1.11	1.05–1.18
Indirect association: Peer problems	1.04	1.01–1.08	1.05	1.02–1.09
Total indirect association	1.18	1.12–1.25	1.19	1.12–1.26
**Male only**				
Total association	2.53	1.80–3.55	1.76	1.22–2.54
Direct association	2.17	1.54–3.06	1.51	1.04–2.18
Indirect association: Internalizing symptoms	1.00	0.97–1.03	1.01	0.97–1.05
Indirect association: Externalizing behaviors	1.08	1.00–1.17	1.06	0.98–1.14
Indirect association: Peer problems	1.07	1.02–1.13	1.09	1.03–1.16
Total indirect association	1.17	1.09–1.25	1.17	1.09–1.25

CI, confidence interval; OR, odds ratio.

a Adjusted for sex, ethnicity, family income, parents in household, alcohol during pregnancy, maternal psychological distress, maternal age, and planned pregnancy or not.

### Complementary analyses

As a complementary analysis, we estimated separate mediation models for males and females. The results showed overall the same patterns of associations as in the whole sample (Table 2). Furthermore, we ran models where smoking was measured as a continuous variable rather than a categorical variable. Results indicate that the odds of suicide attempt increased for each SD increase (i.e. 3.22 cigarettes) in the number of cigarettes smoked (unadjusted OR = 2.46, 95% CI = [2.04, 2.98]; adjusted OR = 2.10, 95% CI = [1.70, 2.58]). Mediation analyses using smoking as a continuous variable yielded similar results to those in the primary analyses (Supplementary Table S2).

## Discussion

Using a longitudinal cohort from the United Kingdom, the objective of this study was to examine the pathways linking maternal smoking during pregnancy to adolescent suicide attempt through childhood internalizing symptoms, externalizing behaviors, and peer problems. Our findings highlight a significant association between prenatal tobacco exposure and increased risks of adolescent suicide attempt, partly explained by childhood behavioral and peer problems from ages 3 to 14 years.

Consistent with previous research, our findings have shown maternal smoking during pregnancy to be a predictor of various psychosocial difficulties in the offspring, suggesting that prenatal nicotine exposure may be associated with neurodevelopmental disruptions in brain regions associated with emotional regulation and inhibitory control (Holz et al., 2014; Wells & Lotfipour, 2023). These disruptions have also been associated with externalizing behaviors, irritability, and difficulties with self-regulation (Cornelius & Day, 2009).

Our study further demonstrates that externalizing behaviors and peer problems are significant predictors of adolescent suicide attempt. These findings are consistent with previous research indicating that early-life emotional dysregulation and social difficulties may increase suicide risk during adolescence (de la Torre-Luque, Essau, Lara, Leal-Leturia, & Borges, 2023; Min et al., 2021). Interestingly, internalizing symptoms in childhood were not significantly associated with suicide attempt in adolescence. This finding contrasts with some prior studies that have found significant associations between internalizing symptoms and later suicidality (Bazrafshan, Sharif, Molazem, & Mani, 2016; Soto-Sanz et al., 2019), but is in line with a Canadian study that found internalizing problems to be associated with suicidal ideation but not suicide attempt in multivariable analyses (Orri et al., 2020).

Our mediation analysis provides further evidence of the indirect pathways through which prenatal smoking increases the risk of adolescent suicide attempt. Externalizing behaviors and peer problems accounted for 12.3% and 9.2% of the association between prenatal smoking exposure and suicide attempt, respectively. This indicates that psychosocial difficulties collectively mediated more than one-fifth of this association. However, the partial mediation suggests that other mechanisms may also contribute to the increased risk of suicide attempts among individuals prenatally exposed to tobacco.

Beyond psychosocial factors, other potential mediators in this association could include birth weight. In fact, birth weight has been found to mediate the association between maternal smoking during pregnancy and later psychiatric symptoms in the offspring (Brannigan, Healy, Cannon, Leacy, & Clarke, 2020). Other potential mechanisms could involve adverse postnatal environments, physical health conditions, or neurodevelopmental alterations. While these factors have been independently associated with both prenatal tobacco exposure and suicidal behaviors in separate lines of research, they have not been systematically investigated as mediators of this association (Ahmedani et al., 2017; Auerbach, Stewart, & Johnson, 2017; Hollis, 1996). Future studies should investigate these additional pathways to provide a more comprehensive understanding of the mechanisms underlying the link between maternal smoking during pregnancy and offspring suicidality. Examining these factors could help clarify the complex interplay between biological, environmental, and developmental influences, ultimately informing more effective prevention and intervention strategies.

Understanding the mediating roles of externalizing behaviors and peer problems has important clinical implications. Evidence-based interventions targeting these psychosocial difficulties may help mitigate the increased risk of suicide attempts among adolescents with prenatal exposure to tobacco. Interventions such as cognitive behavioral therapy or school-based programs have demonstrated effectiveness in reducing externalizing symptoms and in improving peer relationships, as well as global social functioning (Battagliese et al., 2015; Etkin, Juel, Lebowitz, & Silverman, 2023; Ghiroldi, Scafuto, Montecucco, Presaghi, & Iani, 2020). Implementing these interventions early in development, particularly for children identified as having behavioral and social difficulties or who were prenatally exposed to tobacco, could prevent the escalation of these risks and reduce the likelihood of later suicidal behaviors. Our findings also highlight the importance of preventing maternal smoking during pregnancy as a potential strategy for reducing long-term psychosocial and mental health risks in offspring. Public health efforts should thus continue to promote smoking cessation programs targeting expectant mothers, as well as to target risk factors that increase the likelihood of women smoking during pregnancy.

To our knowledge, this is the first study to investigate specific mediating pathways in the association between prenatal tobacco exposure and suicide attempt in adolescence. We used a longitudinal design spanning 17 years, repeated measures of our mediators, and adjusted for several relevant confounding factors. Despite these strengths, some limitations should be acknowledged. To begin, our study does not account for variations in smoking patterns during pregnancy. Differences in the trimesters in which the cigarettes were smoked could have differential effects on child outcomes, since previous studies have shown that early prenatal exposure, particularly during the first trimester, is associated with a significantly increased risk of depressive symptoms in the offspring, whereas exposure later in pregnancy appears to have no significant effect (Duko et al., 2021). The use of self-reported smoking data during pregnancy may also be subject to recall or social desirability bias, potentially leading to inaccurate reporting. Furthermore, while we accounted for key confounders, residual confounding remains a possibility, particularly for unmeasured factors such as paternal smoking during pregnancy or postnatal environmental stressors that may influence both the mediators and the outcome. For instance, unmeasured confounding could arise from maternal psychopathology and personality characteristics not fully accounted for by the Rutter scale. Similarly, we could not account for genetic confounding. Mothers who carry genetic vulnerabilities to mental health or behavioral traits may be more likely to smoke during pregnancy and may also pass on genetic risks that influence their child’s difficulties. This type of genetic correlation, as well as possible gene–environment interactions, could therefore contribute to the documented associations (Thapar & Rice, 2021). Consequently, the associations observed in this study may, at least in part, reflect underlying maternal mental health vulnerabilities rather than a direct effect of prenatal tobacco exposure. A third limitation is that our mediator variables (internalizing symptoms, externalizing behaviors, and peer problems) were parent-reported at each wave of collection. While it ensures consistency across time points and reduces potential biases associated with self-reporting in younger children, it may not fully capture the child’s internal experiences, potentially leading to discrepancies between reported and actual psychosocial difficulties. It can also introduce shared method variance bias, given that smoking behavior was reported by mothers as well. Finally, the fact that we only assessed self-reported suicide attempt, without capturing other suicidal behaviors such as ideation or non-suicidal self-injury, may limit the comprehensiveness of our findings.

In conclusion, our study highlights the significant roles of childhood externalizing behaviors and peer problems in mediating the relationship between prenatal exposure to tobacco and adolescent suicide attempt. Our findings reinforce the need for comprehensive prevention and intervention strategies that address both prenatal risk factors and early-life psychosocial development to reduce the burden of adolescent suicidality.

## Supporting information

10.1017/S0033291725102936.sm001 Bujold et al. supplementary material Bujold et al. supplementary material

## Supplementary material

The supplementary material for this article can be found at http://doi.org/10.1017/S0033291725102936.
