# Supplementary material for: Mediating mechanisms of the association between prenatal tobacco exposure and adolescent suicide attempt: A 17-year longitudinal study
Source: Psychol Med. 2026 Jan 2;56:e6. doi: 10.1017/S0033291725102936 (PMC13092991; doi:10.1017/S0033291725102936)

**Supplementary material**

**Table S1. Sociodemographic characteristics**

|  | **Included (N=8861)** | **Excluded (N=9678)** | **P-value** |
| --- | --- | --- | --- |
|  |  |  |  |
| **Sex** |  |  | < 0.001 |
| Male | 4342 (49%) | 5184 (53.5%) |  |
| Female | 4519 (51%) | 4494 (46.4%) |  |
| Missing | 0 | 0 |  |
| **Ethnicity** |  |  | < 0.001 |
| White | 7160 (80.8%) | 8117 (83.9%) |  |
| Not white (ethnic minority) | 1682 (19%) | 1532 (15.8%) |  |
| Missing | 19 (0.2%) | 29 (0.2%) |  |
| **Family income** |  |  | < 0.001 |
| Low, < 10400£ | 1695 (19.1%) | 3023 (31.2%) |  |
| Medium, 10400 to 31200£ | 4401 (49.7%) | 4529 (46.8%) |  |
| High, > 31200£ | 2046 (23.1%) | 1235 (12.8%) |  |
| Missing | 719 (8.11%) | 891 (9.2%) |  |
| **Presence of caregivers in the household** |  |  | < 0.001 |
| Both parents | 7816 (88.2%) | 7464 (77.1%) |  |
| Blended family or other caregiver | 1045 (11.8%) | 2214 (22.9%) |  |
| Missing | 0 | 0 |  |
| **Maternal alcohol consumption during pregnancy** |  |  | < 0.001 |
| No | 6146 (69.4%) | 7024 (72.6%) |  |
| Yes | 2702 (30.5%) | 2610 (27%) |  |
| Missing | 13 (0.1%) | 44 (0.5%) |  |
| **Maternal psychological distress** |  |  | < 0.001 |
| Mean score | 1.612 | 1.78 |  |
| Missing | 362 (4.1%) | 387 (4%) |  |
| **Maternal age** |  |  | < 0.001 |
| Mean age | 30.01 | 28.3 |  |
| Missing | 6 (0.1%) | 19 (0.2%) |  |
| **Planification of pregnancy** |  |  | < 0.001 |
| Planned | 5295 (59.8%) | 4673 (48.3%) |  |
| Surprise | 3541 (40%) | 4948 (51.1%) |  |
| Missing | 25 (0.3%) | 57 (0.6%) |  |

**Table S2. Total, direct, and indirect associations estimated using smoking as a continuous variable (number of cigarettes), whole sample**

|  | **Unadjusted** | | **Adjusted*** | |
| --- | --- | --- | --- | --- |
|  | **OR** | **95% CI** | **OR** | **95% CI** |
| Total association | 2.46 | 2.04 - 2.98 | 2.10 | 1.70 - 2.58 |
| Direct association | 2.19 | 1.81 - 2.66 | 1.79 | 1.46 - 2.21 |
| Indirect association: Internalizing symptoms | 1.03 | 1.01 - 1.06 | 1.01 | 0.99 - 1.03 |
| Indirect association: Externalizing behaviours | 1.05 | 1.00 - 1.09 | 1.08 | 1.03 - 1.14 |
| Indirect association: Peer problems | 1.04 | 1.01 - 1.07 | 1.07 | 1.03 - 1.10 |
| Total indirect association | 1.12 | 1.08 - 1.17 | 1.17 | 1.12 - 1.22 |

*adjusted for sex, ethnicity, family income, parents in household, alcohol during pregnancy, maternal psychological distress, maternal age, planned pregnancy or not

OR, Odds ratio

CI, Confidence interval

**Figure S1. Missing data in the covariates and mediators.**


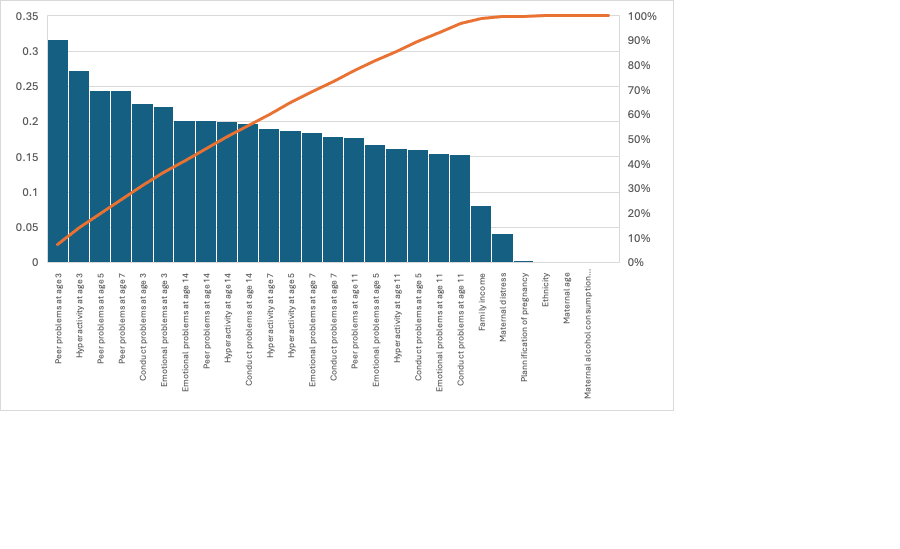

Supplement: Bujold et al. supplementary material [file S0033291725102936sup001.docx]
